# Child Mortality and Nutritional Risks in Rural Chad: A Community-Based Cross-Sectional Study in Béré

**DOI:** 10.3390/ijerph22091320

**Published:** 2025-08-25

**Authors:** Marie-Claire Boutrin, Marci Andersen, Zach Gately, Charis McLarty

**Affiliations:** 1 School of Arts and Sciences, Biological Sciences Department, Oakwood University, 7000 Adventist Blvd NW, Huntsville, AL 35896, USA; 2 School of Public Health, Global Health Department, Loma Linda University, 24951 North Circle Drive, Nichol Hall, Loma Linda, CA 92350, USA

**Keywords:** child mortality, child weight gain, breastfeeding, infectious diseases, vaccination, Sub-Saharan Africa

## Abstract

Chad, a Sub-Saharan country, has some of the worst child mortality and health indicators. A lack of recent and accurate health records in Béré, rural Chad, due to decades of strife compromises the development of relevant health interventions by Project 21, a community health program. This study investigates child mortality, weight gain ability, and related factors in 0–2-year-olds through a cross-sectional study completed by trained community health workers using a survey questionnaire. Most household heads are Nangtchéré Christian males with secondary-level education. Male infants have the highest mortality rate. Infant mortality is predicted by non-exclusive breastfeeding started within 6 days after birth and by the number of household children who experienced and received treatment for meningitis. Toddlers’ mortality is predicted by the number of household adults who experienced and received treatment for meningitis and the number of household adults and children who were vaccinated. The odds of children having no difficulty gaining weight vary with their gender, age, the food or liquid given to them, the source of breastfeeding advice, handwashing and vaccination practices, and experiences with infectious diseases and their treatments. These findings provide targets for future health interventions towards achieving SDG3 in Sub-Saharan Africa.

## 1. Introduction

Child mortality is one of the main health indicators for child health. Undernutrition, malaria, and diarrheal diseases are the top contributors to child mortality for under-fives, especially in the Sub-Saharan African regions [1,2]. The ability of a child to have or maintain a healthy weight is an important indicator of child health worldwide. The inability of a child to gain weight, along with wasting (too thin for their height) and stunted growth (too short for their age), are recognized as components of child growth failure and are associated with future long-term health issues and reduced life expectancy [1,2]. Malnutrition is a common contributor to children’s inability to gain weight and includes undernutrition and inadequate intake of nutrients, vitamins, or minerals [3].

To address these child mortality issues, the Sustainable Development Goal (SDG) 3 includes targets such as (1) ending epidemics of diseases, including malaria, water-borne diseases, and other communicable diseases, and (2) strengthening the capacity of developing countries for early warning and risk reduction by 2030 [4].

In addition, studies have reported the main under-five child mortality determinants in Sub-Saharan Africa to include preventable infections, such as diarrhea, pneumonia, malaria, and HIV/AIDS, as well as malnutrition, hygiene, sanitation, inadequate healthcare infrastructure, poverty, maternal education level, and restricted access to good healthcare standards [5,6]. Exclusive breastfeeding has been promoted by UNICEF for decades to improve child health, and numerous studies have shown it to be a strong predictor of infant survival globally, including in Africa [7,8,9]. Exclusive breastfeeding is reported to provide the nutrition and immune defenses necessary to promote child health in low-income countries, where it is challenging for mothers to adequately feed their children [10]. A study targeting twenty-five Sub-Saharan African countries found that the age, weight, fever or experiences of diarrhea in infants; mothers with higher education level; wealthier households; and location (rural, East, and West Africa) affected the odds of exclusive breastfeeding [9]. Chad, a country found in the center and Sub-Saharan region of Africa, has had poor health indicators for decades [1,11,12]. Although its fertility rate is high, it has alarming child under-five mortality rates (103 deaths/1000 live births), including 32 deaths/1000 live births for newborns and 64 deaths/1000 live births for infants [13,14]. Poverty is prominent in rural areas, resulting in disparities in life expectancy, nutrition, access to healthcare and clean water, and sanitation standards [12,15]. Infectious diseases, as well as maternal, perinatal, and nutritional deficiencies, account for 66% of total deaths in the population, and Chad has one of the highest burdens for child diarrhea and malaria incidence worldwide (26,152/100,000 population) [16,17]. Tuberculosis incidence in Chad is high, and the related mortality rate has increased since 2015 to reach 25/100,000 population in 2021 [16]. Approximately nine neglected tropical diseases, including lymphatic filariasis, are endemic in Chad. While mass drug administration (MDA) interventions in 2020 have helped reduce the incidence rates of four of them, much work is needed to address these diseases [16]. Childhood immunization levels are remarkably suboptimal, with 296,579 children being under-immunized and 190,658 non-vaccinated children, putting children at high risk for vaccine-preventable diseases and related complications, such as outbreaks and death [16]. This data shows the implications of malaria, diarrheal and other infectious diseases, vaccination, sanitation, and nutrition practices for weight gain and child mortality in Chad.

The worst indicators of child mortality and health are found in Tandjilé, a southern region that incorporates several cities, including Béré. The latest national demographic survey revealed that the first-born male children of Baguirmi mothers living north of Béré have the highest mortality risk [18,19]. The national survey also showed that 40% of children experience chronic malnutrition and delayed growth, with 29% of under-fives being notably underweight and only 29% of children receiving diarrhea treatment [18]. Additionally, 98% of children are breastfed but not exclusively, only 6% of the children aged 6–23 months are fed following the recommendations for optimum nutrition, and only 10% of children are treated with anti-malarial drugs [18]. In addition, our recently published socio-demographic study in Béré revealed that the mortality rate for under-fives is a little higher than the national average, worse in infants and toddlers, higher in male children, and influenced by household location, the number of children in households, and whether health information was received from traditional birth attendants (TBAs) [20]. While these statistics call for immediate public health interventions, decades of poverty and deficient administrative infrastructure due to civil strife have hindered governmental action and updates in health records. Although the Chadian government has committed to universal health coverage, challenges such as limited access for residents of rural areas, severe shortages of health care professionals, low funding, high disease burden, health inequities, and a fragile health system have compromised robust developments in the Chadian public health system. Therefore, non-governmental and international entities with health resources, such as Project 21, have been instrumental in promoting health in rural locations such as Béré. Project 21 provides health education, a dental clinic, and community health worker (CHW) and TBA training. While the constitutional order has improved with the recent election of the Chadian president and a new vaccination campaign against malaria, pneumococcal disease, and rotavirus diarrhea has been launched by the Chadian Ministry of Health with the support of Gavi, UNICEF, and WHO, the public health system remains in need of assistance [21].

Future health interventions targeting children’s health in Béré require additional and updated data, which are not available due to ongoing armed conflicts. While the UNICEF guidelines on child feeding (including exclusive breastfeeding) and other child health determinants have been studied globally, the latter and their impact on child mortality and weight gain have not been studied in rural Chadian towns such as Béré and are unknown to date. This study provides novel information on children’s demographics and mortality in the context of their feeding practices (including breastfeeding), ability to gain weight, experiences with infectious diseases, and related factors such as sanitation, handwashing, and vaccination practices in a rural Chadian community setting. To our knowledge, this project is the first public health effort offering this type of health information on this region of Chad. Further, this study reveals possible targets for future health interventions to promote child health in Béré and work towards achieving the aforementioned SDG 3 targets.

## 2. Materials and Methods

### 2.1. Study Setting

The study setting is as described in the first publication from the project [20]. Trained CHWs and Project 21 personnel carried out a study in the twenty-one quartiers of Béré, a southern rural city harboring rice agriculture and farming. The quartier residents were surveyed to gather updated and relevant population data. In addition, village chiefs were included in the planning and preparation of the study to respect the local culture and enable survey implementation. Although respondents seemed happy to participate in the survey, care was exercised during the data collection process to minimize the trigger of feelings such as grief and sadness related to the level of child mortality and diseases encountered in the region.

### 2.2. Survey Respondents

The criteria used to select survey participants and definition of terms have been outlined in the published first portion of the project but are repeated below for the reader’s convenience [20]. Briefly, household heads were targeted for the survey since children were too young to be interviewed. They included all residing ethnicities, religious and socio-economic backgrounds, and males and females aged 18 years and older and are designated as “respondents” in this study. Individuals who were younger than 18 years old, not household heads or not recommended by the household head, mentally unsound or unable to give consent, or who lived less than one year in the household were excluded from participating in the survey. A family unit included one husband, wife/wives, and children; one adult and children; or a single adult. A household included a group of persons who shared the same kitchen or hearth or a group of persons who ate from the same cooking pot/kitchen. Households included a single family (nuclear household) or a combination of cohabiting families (multi-family household). Only one family per multi-family household was interviewed to follow good sampling standards.

### 2.3. Study Design and Sample Size

The details of the methods used for study design and sample size are mentioned in the published first portion of the project but are mentioned below for the reader’s convenience [20]. The study was a community-based cross-sectional survey conducted in the twenty-one quartiers of the rural city of Béré, Chad. The sample size was calculated using the PASS software for a cross-sectional study (https://www.ncss.com/software/pass/, accessed on 1 May 2024) and the expected malaria prevalence in children. Malaria is the top Chadian cause of mortality, especially in the under-five-years-old group, where 41% of children are affected [22]. Sample size determination used a power of 80% (0.8), a desired precision of 5%, and a 95% confidence interval. Using the above parameters, an estimated mean household size of 7, and with 39% of the population being children younger than ten years old, 400 households was determined as the minimum sample size needed to have statistical power for the study [23]. However, additional households were surveyed to promote statistical power.

### 2.4. Data Collection

Data collection was completed as previously described in Boutrin et al. [20]. In this study, the questionnaire was created in English using previously validated questionnaires that investigated demographics, child mortality by age group and gender, breastfeeding and other child nutritional practices, children’s ability to gain weight, and factors known to affect child weight, such as malaria experiences, diarrhea experiences, sanitation practices, and health advice experiences within the last twelve months [18,24,25,26,27,28,29,30]. Diarrhea was defined as the passage of three or more loose or liquid stools per day. The questionnaire was translated and implemented in French. Local CHWs were trained by Project 21 personnel according to WHO guidelines and protocols for teaching and conducting survey data collection, as mentioned in Boutrin et al. [20]. Respondents’ children’s ability to gain weight with no problem was evaluated by visual comparison using aids showing underweight and normal-weight children due to lack of resources in the field. While this method of assessment has been previously used and validated, it may also introduce potential bias due to respondents’ subjectivity [31,32,33,34].

### 2.5. Human Subjects’ Protection

Human subjects’ protection was achieved as described in Boutrin et al. [20]. IRB authorization was secured (IRB approval #5150151), and its guidelines were followed during the project. A verbal consent was read to every person before getting their written consent (if they chose to participate in the study). Data was recorded anonymously to protect respondents’ privacy and confidentiality.

### 2.6. Variables Included in the Study

In this study, the dependent variables include the number of children who died in the last twelve months and the children’s ability to gain weight. Due to limited resources, the ability of children to gain weight was recorded from respondents using pictures of underweight and normal-weight children for comparison with their children’s appearance. The independent variables targeted in the study included factors known to impact child mortality and the ability of children to gain weight, such as breastfeeding and other child feeding practices, exposure to health advice from TBAs and/or CHWs, malaria and diarrhea experiences, and their risk factors (e.g., sanitation practices and conditions, water treatment, vaccination and deworming practices) within the last twelve months. These variables were investigated because (1) previous observations from Project 21 personnel acknowledged underweight children with possible stunting and wasting, breastfeeding mixed with other child feeding practices, diarrhea and malaria outcomes, and sanitation and vaccination issues; (2) official health records were not available for children and mothers; and (3) an understanding of the health context of the children was needed to help address the absence of health records and identify and design future child health interventions in Béré. Data for these variables were collected through respondents’ surveys using the questionnaire.

### 2.7. Data Analysis

The methods, including the software used for data analysis, are described in the previously published first part of the project [20]. Descriptive data analyses, such as frequencies, means, and percentages, were completed to gain understanding of sociodemographic, child mortality, child nutritional practices, weight gain ability, infectious diseases and related factors (e.g., sanitation (handwashing, water treatment), deworming and vaccination practices, and health advice characteristics of the population in Béré. Nutritional practices for children were recorded following two main sources of information: the children’s main age groups outlined by the American Speech–Language–Hearing Association (ASHA) Feeding and Swallowing milestones from 0 to 24 months old and the feeding practices observed in Béré [35,36,37]. This strategy was defined by ASHA, a reputable professional association that provides valid information, and followed its guidelines and included details that made it relevant to the local feeding context. For child mortality data, the age groups used in the study were defined by taking into consideration established pediatric and Centers for Disease Control and Prevention (CDC) age categories and Chadian culture: infant (<12 months old) and toddler (12 to 24 months old) [38,39]. The following formula was used to determine age-specific mortality rates (ASMR): mortality rate per 1000 population = (age-specific number of deaths/age-specific population) × 1000 [40]. Here, the variable “respondents’ children reported dead” is the numerator representing deaths (A), and the “respondents’ children number” stands for the age-specific population (B) (see Table 1). The ASMR per 1000 (C) = ((A)/(B)) × 1000.

Also, One-Way ANOVA was used to compare a continuous variable between three or more groups. The chi-square test of independence was performed to identify associations between categorical variables. In addition, linear regression was used to identify predictors of child mortality (dependent variable), and binomial logistic regression was performed to identify odds ratios of children’s ability to gain weight (dependent variable). The models for the linear and logistic regression studies simultaneously included several independent variables, such as feeding, health advice, handwashing, water treatment, experience and treatment of infectious diseases, deworming, and vaccination practices, all known factors for children’s ability to gain weight and child mortality. Results are considered statistically significant at 5% levels of significance (*p* < 0.05).

## 3. Results

### 3.1. Respondents’ Demographics

The predominant characteristics of the respondents’ population are listed in Table 1: most live in a medium house standard (57.4%), belong to a nuclear family type (78.5%), are evangelical (63.6%), are of Nangtchéré ethnicity (88%), have a secondary school educational level (39.2%), and have a male household head (88.7%).

### 3.2. Child Nutrition Practices

Child nutrition practices are known to be determinants of child health, including weight gain outcomes and mortality. The practices for child nutrition observed in Béré, including breastfeeding, are listed in Table 1 and Table 2. Most of the respondents’ children were breastfed, with almost 62% being breastfed right after birth, 7.1% breastfed one day after birth, and 31.3% breastfed more than one day after birth. In the latter, 91% of the children started breastfeeding three days after birth and 7% two days after birth. The median number of breastfed children was four (range = 18). The highest percentage (95%) of children breastfed right after birth was found in Bolo and Kobtcha. All the respondents from Tchamangue started breastfeeding their children three days after birth. The respondents’ detailed feeding practices of their children can be found in Table 2. While breastfeeding, the children were given liquids for the first time in the first week postpartum and throughout their first six months of life. Water was the predominant liquid given to nearly 89% of breastfed children during their first week of life. During the first and fourth weeks, the main liquid intake, apart from breastfeeding, was milk formula for over 26% of the respondents’ children. From the fifth week to six months after birth, 42–44% of the children were first given other liquids, such as soup (56%), sauce (18%), “bouillon” (broth, 16%), and fruit/vegetables/peanut sauce (8%), while breastfeeding. Also, between the second and sixth month after birth, the children were given thin bouillie (83%)—a watery version of the traditional Chadian “bouillie” dish (cereal paste/flour mixed with water, peanut butter, milk, and sugar)—and juice (62%). From seven to twelve months, a minority (up to 10%) of the respondents’ children were given other liquids, such as juice, soup, bouillon, or sauce, for the first time while breastfeeding. At the toddler stage, similar portions of the children (4%) were given thin bouillie, milk formula, sauce, and thin soups/ bouillon such as thin tomato or potato soup for the first time while breastfeeding.

Concerning solid food intake, nearly two-thirds of the respondents’ children were given their first solid food, mainly bouillie, when aged 2–6 months. Between 7 and 12 months, more than half of the respondents reported that their children were fed other solid foods for the first time—mainly mango, banana, and bouillie.

While respondents reported that a minority (3.5% to 5.9%) of their children stopped being breastfed between 2 and 7 months of age, the majority (90%) of the children stopped being breastfed when they were toddlers.

Health advice and breastfeeding information were received from CHWs and TBAs. Descriptive data on the sources of health advice and breastfeeding information is included in Table 1. Most (92%) of the respondents received health advice from both CHWs and TBAs, with more respondents getting health advice from CHWs (78%) compared to TBAs (65%). Regarding the breastfeeding information, most of the respondents (95%) received information within the last year, and the predominant source of information was the radio (72%). Children of the respondents who received breastfeeding information from TBAs and family were more likely to have no challenges gaining weight, with the family information showing the highest odds (see Table 3). However, children from households that received health advice from TBAs were less likely to successfully gain weight.

### 3.3. Infectious Disease Indicators

Infectious diseases are crucial factors for child health, especially weight gain and mortality, in Sub-Saharan countries such as Chad, with malaria and diarrhea having the highest prevalence. The indicators for malaria experience and treatment recorded in this study are listed in Table 1. Tchamangue had the lowest percentage of respondents (46%) that received treatment after experiencing malaria in their households. Three percent of the respondents did not receive any treatment. Malaria prevalence was statistically associated with location (e.g., Bangar, Bolo, Bere Bornou, Bere Mouraye, Cotton-Tchad, Kobtcha, Nergue Annah, Nergue Bakya, Tchamangue; *p* ≤ 0.04). A One-Way ANOVA test showed that the mean number of cases receiving malaria treatment significantly varied with the respondents’ educational level (F(4, 446) = 4.158, *p* = 0.003). Bonferroni’s post hoc tests for multiple comparisons found that the mean value of treated malaria cases was significantly different between the highest level of education, “baccalaureat +”, and all the other education groups: no education (*p* = 0.002), primary education (*p* = 0.047), secondary education (*p* = 0.013), and baccalaureat (*p* = 0.008).

The indicators for diarrhea are listed in Table 1, including the respondents’ results for adults and children experiencing and receiving treatment for diarrhea. Diarrhea was the predominant infectious disease experienced by adults (93.8%) and children (51.4%) in the last 12 months and one of the best diseases treated in both groups (adults: 93.6%; children: 97.3%). The quartiers with the most diarrhea cases were Béré Mission 1 (combined cases = 98%; treated cases = 90%) and Nergue Goudjiba (combined cases = 94%; treated cases = 92%). Bangar had the lowest diarrhea treatment percentage (combined cases = 92%; treated cases = 72%). Deworming is often used to prevent diarrheal diseases. Most (85%) of the respondents participated in deworming programs in the last 12 months (see Table S1). All the respondents from Béré Bornou, Béré Mission 2, Béré Mouraye, and Nergue Barkya participated in a deworming program in the last twelve months, while those from Nergue-Goudjba had the lowest participation percentage (4.7%). A One-Way ANOVA test showed that the mean number of children with cases of diarrhea that were treated varied statistically significantly with respondents’ educational level (F(4, 446) = 4.263, *p* = 0.002). Bonferroni’s post hoc tests for multiple comparisons found that the mean value of treated diarrhea cases was significantly different between the no education group and primary education (*p* = 0.027) and between no education and secondary education groups (*p* < 0.001).

After diarrhea and malaria, measles was the infectious disease that was most experienced, although to a much smaller extent (adults: 2.2%; children: 5.5%), and it was well treated (adults: 100%; children: 92.2%). The next noticeable disease was tuberculosis (adults: 2.9%; children: 1.7%), which was also well treated (adults: 92.3%; children: 91.7%). Meningitis was twice as high in children than in adults (adults: 1.3%; children: 2.7%) but not well treated (adults: 33.3%; children: 60.3%). Adults were reported to have more cases of lymphatic filariasis than children (adults: 1.8%; children: 0.2%), and all cases were reportedly treated. Dole was the quartier with the highest meningitis and measles prevalence; Tchouroue-Yendei had the most cases of tuberculosis and pneumonia; and Béré Bornou and Béré Poste showed the highest prevalence of rabies and lymphatic filariasis.

Vaccination is preventative against infectious diseases. The percentages of vaccinated children are reported in Table 1 and are generally lower than those for adults for all vaccines in Béré. The BCG, VAT1, and VAT2 vaccines showed the highest percentage of participation for both adults and children, whereas VAT4 had the lowest percentage of participation in both groups.

### 3.4. Handwashing Practices

Descriptive data on handwashing are available in Table 1. Most (98%) respondents report washing their hands. More respondents washed their hands with water only (68%), followed by water and soap (55.7%) after using the toilet. Compared to ‘sometimes’, twice as many respondents ‘always’ washed their hands with water and soap after using the toilet. Additionally, more respondents washed their hands before eating with water and soap (73.6%) than with water only (55.4%). The majority (91%) of those who washed their hands with water before eating ‘always’ applied this practice. After toilet use, 90% of the respondents from Nergue Bakya ‘always’ washed their hands with water, whereas those from Ivergue Touichiroue, Toupadjre Mbassea, and Cotton-Tchad mostly implemented the practice ‘sometimes’. The quartiers with the highest soap handwashing practices were Nergue Bakya, Béré Bornou, Singuir, and Cotton-Tchad. The respondents from Cotton-Tchad, Toupadjre Mbassea, and Toupadjer Ngolo showed top water handwashing practices before eating, and those from Béré Mouraye, Nergue Bakya, and Toupadjer Ngolo had the highest soap handwashing practices.

### 3.5. Respondents’ Children Having No Problem Gaining Weight

Descriptive data on the respondents’ children’s ability to gain weight are included in Table 1. Approximately 29% of the respondents stated their children had problems gaining weight, while the rest (71%) did not. Population characteristics such as multi-families, Arabics, Muslims, no educational level, received breastfeeding information within the last 12 months, “TBA did not give information”, “family did not give breastfeeding information”, and “radio gave breastfeeding information” had the highest proportion (40.2%, 71.4%, 55.6%, 49.4%, 29.5%, 35.3%, 31.9%, and 32.8%, respectively) of respondents with children having problems gaining weight. The quartiers with the highest percentages of respondents with children having difficulty gaining weight were Bolo (90%) and Béré Mouraye (79%), while all respondents residing in Cotton-Tchad, Ivergue Touichiroue, Tcha-Asse, Tchamangue, and Yendei reported their children had no issues gaining weight. Chi-square tests of independence reveal that the association between quartiers and children having no problem gaining weight is significantly linked to house standard (low standard (χ^2^(16, N = 68) = 37.481, *p* < 0.001), medium standard (χ^2^(20, N = 259) = 101.145, *p* < 0.001), high standard (χ^2^(17, N = 124) = 45.658, *p* < 0.001)), respondents’ educational level (no education (χ^2^(20, N = 85) = 57.234; *p* < 0.001), primary school level (χ^2^(17, N = 127) = 38.381, *p* = 0.002), secondary school level (χ^2^(20, N = 177) = 70.522, *p* < 0.001)), household head male gender (χ^2^(20, N = 400) = 149.414; *p* < 0.001), and water being treated (χ^2^(20, N = 394) = 147.419; *p* < 0.001).

The factors associated with the respondents’ children’s ability to gain weight were investigated using logistic regression, and the statistically significant results are shown in Table 3. Regarding feeding practices, boys aged 2–6 months old who received thin bouillie (liquid) for the first time while being breastfed were 1.3 times more likely to have no difficulty in gaining weight (*p* = 0.020). Female toddlers who were fed bouillie as solid food for the first time were 42% (*p* = 0.030) less likely to have no problem gaining weight. The children who started breastfeeding one day after birth were 2.6 times more likely to have no issues gaining weight (*p* = 0.029), and those who started breastfeeding more than one day after birth were 25 times more likely to have no difficulty gaining weight (*p* < 0.001), compared to those who started right after birth. The children of the respondents who received breastfeeding information from TBAs were almost twice as likely to have no issues gaining weight (*p* = 0.016), and the children of those who received breastfeeding information from family were over ten times more likely to have no problems gaining weight (*p* = 0.004). However, the children of the respondents who obtained breastfeeding advice from the radio were nearly 50% less likely to do so (*p* = 0.021).

Regarding sanitation and hygiene, children from households who treated their drinking water were 69% less likely to have no challenges gaining weight (*p* = 0.005), compared to those who did not treat it. Handwashing practiced after using the toilet “always with water and soap” lowered the odds of children having no difficulty gaining weight by 71% (*p* < 0.001). However, practicing handwashing before eating “always with water” or “sometimes with water and soap” increased the children’s odds of having no challenges gaining weight (compared to the absence of each respective handwashing practice; *p* < 0.001 for both).

Regarding infectious diseases, the number of household children who had rabies infections or received treatment for rabies in the last twelve months decreased the odds of children having no difficulty gaining weight by 96% (*p* = 0.021 and *p* = 0.018, respectively). Also, the number of household children who were treated for meningitis in the past twelve months considerably (60%) lowered the likelihood of the respondents’ children having no challenges gaining weight (*p* = 0.018). Furthermore, children from households with adults and children who received VAA and BCG immunizations and adults with VAT1 vaccination in the past three years were more likely to have no challenges gaining weight (*p* = 0.001, *p* = 0.018, *p* = 0.002, *p* = 0.006, and *p* < 0.001, respectively). However, children from households who had children vaccinated with VAR in the last three years show decreased (26% less) odds of having no difficulty gaining weight (*p* = 0.030).

### 3.6. Child Mortality Indicators

The ASMRs are outlined in Table 1. The ASMR for infants is the highest (almost three times that of toddlers), with the one for male infants being the worst (343.75 per 1000). The ASMRs are worse for the boys compared to the girls, regardless of the age group.

Multiple variable linear regression analysis was conducted to identify predictors of child mortality in Béré. All the statistically significant results are listed in Table 4.

Regarding feeding practices, a statistically significant model for female infant mortality (F(10, 440) = 6.424, *p* < 0.001, adjusted R^2^ = 0.108) is obtained when giving thin bouillie (liquid) to 0–6-day-old girls (*p* < 0.001) for the first time while breastfeeding. Also, giving bouillie as the first solid food to 0–6-day-old males is shown to predict infant mortality (F(9, 441) = 11.784, *p* < 0.001, adjusted R^2^ = 0.177).

Concerning infectious diseases and child mortality, male and female infant mortality can be predicted by the number of household children who had meningitis in the last twelve months (F(7, 443) = 6.788, *p* < 0.001, adjusted R^2^ = 0.083 and F(7, 443) = 16.226, *p* < 0.001, adjusted R^2^ = 0.191, respectively). Regarding toddlers, the number of experiences and treatments of meningitis in household adults in the last twelve months are significant predictors for male (F(6, 444) = 10.370, *p* < 0.001, adjusted R^2^ = 0.111 and F(6, 444) = 109.737, *p* < 0.001, adjusted R^2^ = 0.592) and female (F(6, 444) = 6.080, *p* < 0.001, adjusted R^2^ = 0.063 and F(6, 444) = 37.794, *p* < 0.001, adjusted R^2^ = 0.329) mortality. Also, male toddlers’ mortality can be predicted by the number of household adults who received VAT4 and BCG vaccinations (F(7, 443) = 7.759, *p* < 0.001, adjusted R^2^ = 0.095) and the number of household children who had BCG, VAT4, and VAR vaccinations (F(7, 443) = 7.826, *p* < 0.001, adjusted R^2^ = 0.096) in the last three years. Female toddlers’ mortality can be predicted by the number of household adults and children who received BCG vaccination (F(7, 443) = 5.102, *p* < 0.001, adjusted R^2^ = 0.060 and F(7, 443) = 3.766, *p* < 0.001, adjusted R^2^ = 0.041, respectively) in the last three years. The number of household children vaccinated with VAR in the last three years is negatively associated with child mortality.

### 3.7. Covariate Analysis

Additional analyses were completed to identify the existence of relationships between covariates. Chi-square tests of independence evaluating relationships between categorical covariates show significant associations between respondents’ religion and educational level (χ^2^(24, N = 451) = 68.540; *p* < 0.001), ethnicity and educational level (χ^2^(20, N = 451) = 54.102; *p* < 0.001), household head gender and educational level (χ^2^(4, N = 451) = 43.709; *p* < 0.001), age and religion (χ^2^(24, N = 451) = 68.540; *p* < 0.001), and age and educational level (χ^2^(16, N = 451) = 34.338; *p* = 0.005).

## 4. Discussion

Chad, one of the Sub-Saharan countries, is known to have some of the worst child mortality and child health indicators, such as malnutrition, underweight children, stunting, and wasting, all known risk factors for child mortality. This study provides valuable information on the feeding practices of children and the factors affecting child mortality and weight gain (e.g., sanitation, infectious diseases, and handwashing) in Béré, a southern rural district, that will enable the development of interventions towards the goals of the SDG 3 for 2030, especially for under-five-year-old children.

First, the study’s respondents’ child mortality indicators, including mortality rates, are higher than Chadian national levels, as observed in our previous study on the socio-economic factors associated with child mortality in Béré [20]. Sociodemographic determinants are also associated with the relationship between children having no problem gaining weight and quartiers: all house standards, respondents with no education, primary and secondary school educational levels, and male household heads. Therefore, Béré residents with these sociodemographic characteristics should be targeted in future health investigations to gain an understanding of the possible practices contributing to these connections.

Second, our study reveals the absence of exclusive breastfeeding in Béré, which agrees with the latest national health survey and other reports showing very low to non-existent exclusive breastfeeding practices in Chad, especially in rural women [18,41,42]. Our results show that the lack of exclusive breastfeeding starting within 6 days after birth is a predictor of infant mortality in Béré and should be a target for future health interventions. These results support previous reports and may reflect Chadians’ beliefs, especially in rural regions where traditional beliefs and practices perceive exclusive breastfeeding as insufficient and detrimental to infants’ health [9,42]. The types of liquid and solid foods given to children while breastfeeding lack variety and nutrients, such as iron and zinc, and create an essential need for food fortification or use of animal-derived foods, as observed in previous reports [18,43]. Liquids used to complement or feed the babies before breastfeeding in Chad were found to be likely contributors of malnutrition and problems in weight gain in children through disruption of digestive tract equilibrium, diarrhea onset, and nutritional deficiencies [18,43]. Ways to alleviate these practices should be considered and incorporated into future health interventions. In addition, the children without early initiation and breastfed after at least 1 day also show higher odds of having no difficulty gaining weight. These findings portray local beliefs that withholding colostrum from newborns and starting breastfeeding at least one or a few days after birth promote the baby’s health. These findings do not align with UNICEF recommendations for young child feeding, where infants should be put to breast within one hour of birth and exclusively breastfed for the first 6 months of life to promote child health [10,41,42]. These results vary from previous studies and suggest that a universal breastfeeding approach may not impact child health outcomes the same way in Béré where the lifestyle and cultural context are different. These observations should be taken into consideration to make relevant future health recommendations. The above difference in results could also be due to potential bias related to the method used to assess children’s difficulty gaining weight: a visual comparison using photos of underweight and normal-weight children, which was used because of lack of resources in the field at that time. The cost of purchasing and shipping equipment to Béré, as well as the uncertainty of receiving shipped items and the lengthy delivery time associated with that location, contributed to the challenges of securing the routine equipment for children’s weight measurements. Respondents may have been uncomfortable admitting that their children were having problems gaining weight—because of pride or subjectivity or recall inaccuracy—thus introducing potential bias. Therefore, future interventions integrating anthropometric measurements are recommended to gain measurements of weight and nutritional status of children, and fundraising campaigns should be planned to facilitate the availability of professional equipment in the field. Also, our results include decreased odds of having no difficulty gaining weight when the first bouillie (solid food) is given during the toddler stage. WHO and UNICEF recommend giving infants their first solid food when they are at least seven months old when exclusive breastfeeding is not practiced, and waiting until the toddler age rather than seven months old may hinder weight gain in children. As a result, the WHO and UNICEF dietary recommendations should be encouraged in future health interventions in Béré, since they do not practice exclusive breastfeeding [44].

Third, infectious diseases were investigated, as they are known risk factors for child mortality and weight gain. Our results reveal that Tchamangue had the lowest percentage of respondents receiving malaria treatment. Considering the results of our previous study on the socio-demographic characteristics of Béré, where households from Tchamangue have primary school (low) education levels, 25% of uneducated members, and an average of nine members and seven children, it is possible that the high number of household members, especially children, causes a financial strain, and thus, malaria treatment cannot be obtained for all household members [44]. Another possibility would be that the household members are so involved with their daily occupation that they do not have the time to receive treatment, or there is a lack of knowledge on the need to receive adequate treatment for malaria due to low education level. Also, the study shows that malaria prevalence is associated with location, suggesting that malaria experiences are not the same in all the quartiers. This may be due to the variety of malaria-related practices found in the quartiers of Béré (e.g., water storage, mosquito bite prevention, mosquito control), the perception of malaria as a health threat, and the related behaviors displayed towards malaria prevention, such as the use of mosquito nets, body and house repellents, and clothing choices. In addition, Bangar has the lowest percentage of diarrhea treatment, which may be due to difficulty in accessing diarrhea treatment (e.g., time outside of daily occupation, distance to hospital, or medical help), the entire household being engaged in daily employment (0% household unemployment) leaving no time for sick treatment, and the perception of diarrhea as not a significant health threat [44]. The above possibilities should be explored in future health studies to gain a deeper understanding of health determinants in Béré. Furthermore, our findings identify adults experiencing and having received treatment for meningitis as child mortality predictors and the number of household children who received treatment for meningitis decreasing the children’s likelihood of having no problem gaining weight in Béré. These observations could reflect the outcomes of meningitis transmission in the household, such as the infection of household children and subsequent sickness, or a decrease in children feeding events due to parents’ illness. Meningitis symptoms include loss of appetite, nausea, vomiting, lethargy, and fever, which are all possible drivers of lower food intake and nutrient absorption, resulting in weight loss. Meningitis treatment could improve those symptoms, but weight gain may take time to occur, especially in an economically challenged environment such as a rural region in Chad. The involvement of meningitis in child mortality and health issues in Béré is not surprising and is congruent with the literature. Previous studies have reported Chad to belong to the Sub-Saharan meningitis belt where babies and young children are at greater risk of infection, and meningitis community outbreaks and epidemics are still occurring, leading to death or long-term sequelae [45,46]. In addition, the number of household children with rabies infections and treatment decreases the odds of 0–2-year-olds not having issues gaining weight by 96%, which is considerable and higher than national levels [16]. The main predictor of rabies in Chad is free-roaming domestic dogs, and future interventions should consider these factors to promote rabies control and promote child health outcomes [47]. Our findings confirm the high burden of infectious diseases in Chad and its role in under-fives’ deaths and weight gain issues [16]. Along with previous reports, this study supports the relevance of preventative strategies, such as vaccination campaigns and animal control, against infectious diseases in Béré and the need to improve infant care, the age group with the highest mortality rate [16,48].

Fourth, since vaccination and sanitation affect the burden of infections in communities, they were also explored in this study. Household vaccination adherence within the last 3 years with VAR, VAT4, and BCG for adults and children significantly predicts child mortality, and VAT1 in adults, VAA and BCG in adults and children, and VAR in children affect the odds of children gaining weight in Béré. While VAR is negatively associated with child mortality, the number of household children vaccinated with it decreases the odds of respondents’ children gaining weight without difficulty. Further investigation should be conducted on child health status and practices following the first dose of the VAR vaccine and whether children receive the second dose of the vaccine at the recommended time to identify factors affecting the children’s ability to gain weight. Therefore, vaccination access and adherence should be included in the strategies to promote health and improve suboptimal children vaccination levels observed in Béré and throughout the country [48]. Additionally, this study shows that sanitation adherence through water treatment and handwashing practices, especially before eating, significantly influences the odds of children having no problem gaining weight and predicts their mortality. Previous reports show that microbial contamination and transmission are hindered by good handwashing practices, including soap rather than just water before eating, and good sanitation practices such as water treatment [18,27,49,50,51,52]. Therefore, obtaining decreased odds of children having no problem gaining weight when the water is treated is unusual and may reflect issues with water treatment. Future studies to investigate the types of water treatment used in Béré are recommended to confirm the efficiency of the treatment processes used. Furthermore, the observation that the likelihood of children having no problem gaining weight decreases considerably when hands are washed “always” with water and soap after using the toilet do not align with previous reports, and possible explanations are that (1) although soap and water are used, the way hands are washed is not adequate and efficient (e.g., time spent washing hands, areas washed, etc.), or (2) the soap may be old or dirty, with its cleaning properties diminished or lost. This reinforces the fact that the cleaning effectiveness of soap is contingent on its actual disinfecting properties and the practice of adequate handwashing. Our results bring awareness to the need for additional education and training in water treatment and handwashing, especially handwashing after using the toilet, where microbial contamination risk is high and may increase the risk of developing diarrheal diseases. The development of resources facilitating efficient water treatment and handwashing with adequate soap and water in private and public toilets is also recommended. Diarrheal disease is the main cause of weight loss, dehydration, malnutrition, and death in under-fives and was found to be a child mortality predictor in Béré and in previous studies [18,27,49,50,51,52]. Therefore, interventions improving water quality and handwashing facilities with water and soap are actively required to lower diarrheal disease prevalence and improve children’s odds of weight gain and survival.

Fifth, some differences in child mortality predictors between genders are apparent in our findings. Gender inequalities in child mortality outcomes have also been observed in previous studies and have been connected to biological, psychological, and social differences between genders or reflections of patriarchal and sexist ideologies, where girls’ lives are perceived as less valuable than boys’ [53]. Higher under-five mortality in boys can be due to biological and genetic differences, where boys are biologically weaker and thus, more vulnerable to premature death [54]. While X-linked immunoregulatory genes may enable higher female resistance to infectious diseases, environmental challenges, such as inadequate diets and difficulties accessing healthcare, can contribute to increased female child mortality [55]. The Gender Inequality Index is also associated with increased girls’ mortality in early life, especially in low-income countries, and preconception and prenatal environmental factors are hypothesized to explain the mortality differences between genders [53,56]. In addition, low-income countries have been found to likely display a gender bias in reporting under-five mortality, where female deaths are under-reported because families favor reporting male deaths [53,57]. Since the regression unstandardized B value for the child mortality predictors in this study is higher for feeding practices for 0–6-day-old-male infants, as well as for male toddler mortality associated with meningitis experiences and treatment and vaccination programs participation, it is likely that male infants and toddlers have a greater association with child mortality when experiencing these factors. These findings may be due to a greater biological and genetic vulnerability of male infants and toddlers in Béré and/or under-reporting of female infants’ and toddlers’ mortality, as observed in other patriarchal low-income countries. These results also show the complexity in identifying the causes of gender inequalities for infant and toddler mortality in Béré and emphasize the need to further investigate the contributors of child mortality in that location.

Sixth, our results show that breastfeeding advice by TBAs and family increases the children’s odds of gaining weight (in contrast to advice from the radio), confirming the importance of adequate breastfeeding education in child health. As previously mentioned, early and exclusive breastfeeding with consideration to the local practices that are found beneficial in this study are crucial to child and maternal health. Early breastfeeding provides a source of natural antibodies to the baby that offers protection against infections, reducing infant deaths caused by diarrheal diseases and lower respiratory tract infections [18,43,58]. It also helps the production of maternal milk and oxytocin, which helps with uterus contraction after childbirth and the ejection of milk [58]. Therefore, a lack of breastfeeding information may deprive the mother and the baby of these benefits, subjecting them to low milk production and increased vulnerability to pathogens. Since TBAs’ advice showed lower odds in children gaining weight than family, expansion in training of TBAs should be implemented to promote the dissemination of beneficial information. Future interventions promoting exclusive breastfeeding practices through trained TBA interventions should be considered crucial for improvements in child and maternal health. This strategy was successful with rural women in Ethiopia and confirms the importance of incorporating one-to-one contact between health personnel and women of reproductive age in educational programs to promote the initiation and prevalence of breastfeeding [58]. The provision of emotional support mechanisms for families experiencing bereavement due to the notable level of child mortality encountered in Béré should also be considered.

Lastly, the results from this study can be used as informative tools to advocate for new policies and guidelines from Chadian public health entities. It is recommended that qualified personnel from Project 21 connect with the health and political representatives for Béré and the Tandjilé region to present the findings from the Béré project (including this study) to the residents of Béré, since they are the targeted population. Based on the results, it is suggested that a revision of the health policies and awareness campaigns for the above locations be conducted. These policies should be inclusive and comprehensive and able to reach rural regions to promote the health of rural communities such as Béré. Collaborative efforts from public health and government officials, local stakeholders, and community representatives, including village chiefs, are requested to promote success.

### Limitations of the Study

While efforts were made to conduct a strong study, it may, however, present limitations related to respondents’ involvement, such as a potential for reporting bias due to inaccurate recall, misunderstanding of the questions, or attempts to give answers perceived to be acceptable to interviewers or politically correct. Also, errors may have occurred during data entry. Unequal distribution of respondents for the quartiers may also affect the confidence intervals’ range. In addition, the method used to assess children’s ability to gain weight was based on visual comparison using aids showing underweight and normal-weight children. While this method was used as a starting point because of the lack of proper resources in the field and provided a level of information, it may also introduce potential bias due to respondents’ subjectivity and pride. Lastly, while our findings concern a rural population, they may not reflect the circumstances of other rural communities and should be extended to other projects with caution.

## 5. Conclusions

This study offers an understanding of the factors affecting child mortality in the context of child-feeding practices, weight gain, and related known risk factors in Béré, Chad, all relevant targets for the SDG3 in Sub-Saharan Africa. Our findings provide insight into gender- and age-related indicators and nutrition practices for infants and toddlers. Predictors of child mortality included non-exclusive breastfeeding practices, experiences and treatment of meningitis in household adults and children in the last 12 months, and VAR, VAT4, and BCG immunization adherence in household adults and children in the last 3 years. Factors affecting the respondents’ children’s odds of not having challenges gaining weight included non-exclusive breastfeeding practices, timing of breastfeeding start, water treatment and handwashing practices, rabies and meningitis experiences and treatment of households’ children, vaccination with VAT1, VAT2, VAA, and BCG of household adults and children in the last 3 years, and breastfeeding information sources. Taken together, our findings warrant future health interventions focusing on breastfeeding initiation that reflect this study’s results: the promotion of exclusive breastfeeding practices, improved access to soap and clean water and education on efficient handwashing, vaccination access and adherence, preventive strategies for meningitis and rabies, and expanded training of TBAs. These interventions are crucial to make the much-needed progress towards the SDG3 for Sub-Saharan Africa.

## Figures and Tables

**Table 1:** Demographic characteristics of respondents and their reported children.

Characteristics	Item	Count (%)	
**Respondents (*n* = 451)**			
Household type	Nuclear family	354 (78.5)	
	Multi-family	97 (21.5)	
Religion	Seventh Day Adventist	19 (4.2)	
	Evangelical	287 (63.6)	
	Muslim	9 (2.0)	
	Catholic	111 (24.6)	
	Animist	9 (2.0)	
	Other	7 (1.6)	
	No religion	9 (2.0)	
Ethnicity	Ngambay	39 (8.6)	
	Nangtchéré	397 (88.0)	
	Fulani	2 (0.4)	
	Arabic	7 (1.6)	
	Other	6 (1.3)	
House standard	Low	68 (15.1)	
	Medium	259 (57.4)	
	High	124 (27.5)	
Age group	<20 years old	1 (0.2)	
	20–29 years old	66 (14.6)	
	30–39 years old	158 (35.0)	
	40–59 years old	191 (42.4)	
	60+ years old	35 (7.8)	
Education level	None	85 (18.8)	
	Primary	127 (28.2)	
	Secondary	177 (39.2)	
	Baccalauréat	33 (7.3)	
	Baccalauréat +	29 (6.4)	
Education period	Number of years	mean = 8.3	
Household head gender	Male	400 (88.7)	
	Female	51 (11.3)	
Household size	Number of members	mean = 7.1	
Received health advice	*From CHW/TBA*		
	Yes	414 (91.8)	
	No	37 (8.2)	
	*From CHW only*		
	Yes	350 (77.6)	
	No	101 (22.4)	
	*From TBA only*		
	Yes	294 (65.2)	
	No	157 (34.8)	
Received breastfeeding advice	*Within the last 12 months*		
	Yes	430 (95.3)	
	No	21 (4.7)	
	*From CHW*		
	Yes	266 (59.0)	
	No	185 (41.0)	
	*From TBA*		
	Yes	284 (63.0)	
	No	167 (37.0)	
	*From family*		
	Yes	53 11.8)	
	No	398 (88.2)	
	*From radio*		
	Yes	323 (71.6)	
	No	128 (28.4)	
	*Other sources*		
	None	448 (99.3)	
	Hospital	1 (0.2)	
	Physician	1 (0.2)	
	Neighbor	1 (0.2)	
Time of breastfeeding start	Right after birth	278 (61.6)	
	1 day after birth	32 (7.1)	
	More than one day after birth	141 (31.3)	
Problem with child gaining weight	Yes	129 (28.6)	
	No	322 (71.4)	
**Respondents’ children aged 0–24 months old (*n* = 315)**			
Children’s gender	Male	178 (56.5)	
	Female	137 (43.5)	
Children number	<12 months old (infant)	113 (35.9)	
	12–24 months old (toddler)	202 (64.1)	
Children reported dead	<12 months old (infant)	37 (59.7)	
	12–24 months old (toddler)	25 (40.3)	
**Mortality rates**			
*Age*	*Population*	*Number of deaths*	*ASMR* *per 1000*
<12 months old (infant)	113	37	327.43
Male	64	22	343.75
Female	49	15	306.12
12–24 months old (toddler)	202	25	123.76
Male	114	15	131.57
Female	88	10	113.63

ASMR: age-specific mortality rate.

**Table 2:** Respondents’ child-feeding practices per age group.

Percentage of Children per Age Group When They Were First Given Liquids While Breastfeeding												
Age group	0–6 days		1–4 weeks		5–7 weeks		2–6 months		7–12 months		13–24 months	
Gender	Male	Female	Male	Female	Male	Female	Male	Female	Male	Female	Male	Female
Water	46.2	42.3	0.04	0.04	0.5	0.8	4.8	4.2	0.2	0.1	0.3	0.3
Juice	1.8	0.7	3.0	4.2	7.5	7.8	30.7	31.9	5.5	5.0	0.7	0.7
Milk	12.8	12.4	13.7	12.6	0.9	3.0	16.8	19.9	2.9	1.3	2.2	1.1
Thin bouillie	2.5	2.8	0.2	0.3	1.2	1.0	44.0	39.2	2.6	2.1	1.8	2.2
Other	0.0	0.7	0.2	0.0	21.5	20.7	21.9	22.4	4.1	4.1	2.2	1.9
**Percentage of children per age group when they were given their first solid food/bouillie**												
Age group	0–6 days		1–4 weeks		5–7 weeks		2–6 months		7–12 months		13–24 months	
Gender	Male	Female	Male	Female	Male	Female	Male	Female	Male	Female	Male	Female
Bouillie	1.6	2.0	0.09	0.04	0.4	0.5	34.9	31.0	11.4	10.7	3.4	3.8
Other	0.0	0.0	0.0	0.0	0.9	1.6	11.6	11.3	30.3	26.3	8.7	9.2
**Percentage of children per age group when breastfeeding was stopped**												
Age group	0–6 days		1–4 weeks		5–7 weeks		2–6 months		7–12 months		13–24 months	
Gender	Male	Female	Male	Female	Male	Female	Male	Female	Male	Female	Male	Female
Child number	0.4	0.4	0.0	0.0	0.0	0.0	1.4	2.1	2.9	3.0	47.3	42.4

**Table 3:** Statistically significant odds ratios for factors associated with children having no problem gaining weight.

	Odds Ratio	95% CI	Adjusted *p*
**Age and gender when first given liquids while breastfeeding**			
2–6-month-old male (thin bouillie)	1.27	1.09–1.47	0.020
**Age and gender when given first solid food**			
13–24-month-old female (bouillie)	0.58	0.38–0.90	0.030
**Time when breastfeeding started**			
1 day after birth	2.63	1.102–6.29	0.029
More than 1 day after birth	25.26	9.08–70.21	<0.001
Right after birth (Reference)	-	-	-
**Water treated**			
Yes	0.31	0.138–0.708	0.005
No (Reference)	-	-	-
**Received breastfeeding advice from**			
Traditional birth assistant	1.87	1.17–2.97	0.016
Family	10.58	2.50–44.79	0.004
Radio	0.52	0.30–0.88	0.021
**Handwashing practice**			
After using the toilet: With water and soap, always	0.29	0.159–0.519	<0.001
After using the toilet: No washing with water and soap (Reference)	-	-	-
Before eating: With water, always	4.81	2.68–8.63	<0.001
Before eating: No washing with water (Reference)	-	-	-
Before eating: With water and soap, sometimes	10.60	4.16–27.01	<0.001
Before eating: No washing with water and soap (Reference)	-	-	-
**Infectious diseases**			
Number of household children with rabies in last 12 months	0.04	0.005–0.34	0.021
Number of household children treated for meningitis in last 12 months	0.40	0.21–0.76	0.018
Number of household children treated for rabies in last 12 months	0.04	0.005–0.33	0.018
**Vaccination**			
Number of household adults with VAT1 vaccine in last 3 years	2.62	1.69–4.07	<0.001
Number of household adults with VAA vaccine in last 3 years	5.15	2.11–12.55	0.001
Number of household adults with BCG vaccine in last 3 years	1.78	1.27–2.48	0.002
Number of household children with BCG vaccine in last 3 years	1.32	1.12–1.56	0.006
Number of household children with VAA vaccine in last 3 years	1.46	1.12–1.91	0.018
Number of household children with VAR vaccine in last 3 years	0.74	0.58–0.94	0.030

**Table 4:** Statistically significant feeding practices and other factors predicting child mortality.

	Unstandardized B	95% CI for B	Adjusted *p*
**Infants’ mortality** *Age and gender when first given liquids while breastfeeding*			
0–6-day-old females (thin bouillie)	0.212	0.116–0.308	<0.001
*Age and gender when given first solid food*			
0–6-day-old male (bouillie)	0.444	0.268–0.620	<0.001
*Infectious diseases*			
For males			
Number of household children with meningitis in last 12 months	0.131	0.091–0.171	<0.001
For females			
Number of household children with meningitis in last 12 months	0.276	0.224–0.327	<0.001
**Toddlers’ mortality**			
*Infectious diseases*			
For males			
Number of household adults with meningitis in last 12 months	0.401	0.300–0.503	<0.001
Number of household adults treated for meningitis in last 12 months	1.988	1.835–2.141	<0.001
For females			
Number of household adults with meningitis in last 12 months	0.202	0.132–0.271	<0.001
Number of household adults treated for meningitis in last 12 months	0.990	0.859–1.120	<0.001
*Vaccination*			
For males			
Number of adults with VAT4 vaccine in last 3 years	0.072	0.023–0.121	0.014
Number of adults with BCG vaccine in last 3 years	0.048	0.020–0.076	0.005
Number of children with BCG vaccine in last 3 years	0.020	0.007–0.033	0.014
Number of children with VAT4 vaccine in last 3 years	0.029	0.009–0.049	0.014
Number of children with VAR vaccine in last 3 years	−0.031	−0.055–(−0.007)	0.026
For females			
Number of adults with BCG vaccine in last 3 years	0.044	0.025–0.063	<0.001
Number of children with BCG vaccine in last 3 years	0.019	0.010–0.028	<0.001

## Data Availability

The dataset generated by the survey research and/or analyzed during the current study is available in the Zenodo repository, 10.5281/zenodo.15782489.

## Supplementary Materials

The following supporting information can be downloaded at https://www.mdpi.com/article/10.3390/ijerph22091320/s1, Table S1: Supplemental demographic information.

## Abbreviations

The following abbreviations are used in this manuscript:

SDG3	Sustainable Development Goal 3
TBA	Traditional birth attendant
CHW	Community health worker
CDC	Center for Disease Control and Prevention
WHO	World Health Organization
ASHA	American Speech–Language–Hearing Association
ASMR	Age-specific mortality rate
VAT	Tetanus toxoid vaccine
VAR	Varicella vaccine
VAA	Yellow fever vaccine
BCG	Bacille Calmette–Guérin (tuberculosis) vaccine
