# Supplementary material for: Child Mortality and Nutritional Risks in Rural Chad: A Community-Based Cross-Sectional Study in Béré
Source: Int J Environ Res Public Health. 2025 Aug 25;22(9):1320. doi: 10.3390/ijerph22091320 (PMC12469488; doi:10.3390/ijerph22091320)
Supplement: Supplementary file 1 [file ijerph-22-01320-s001.zip › ijerph-3762890-supplementary.pdf]

Table S1. Supplemental demographic information

| Characteristics | Item | Count (%) |
|---|---|---|
| **Respondents (n=451); Respondents' children aged 0-10 years old (n=2,104)** | | |
| Hand washing | Yes | 444 (98.4) |
| | No | 7 (1.6) |
| | *After using toilet- With water* | |
| | Always | 155 (34.4) |
| | Sometimes | 154 (34.1) |
| | No | 142 (31.5) |
| | *After using toilet- With water and soap* | |
| | Always | 173 (38.4) |
| | Sometimes | 78 (17.3) |
| | No | 200 (44.3) |
| | *Before eating- With water* | |
| | Always | 231 (51.2) |
| | Sometimes | 19 (4.2) |
| | No | 201 (44.6) |
| | *Before eating- With water and soap* | |
| | Always | 174 (38.6) |
| | Sometimes | 158 (35.0) |
| | No | 119 (26.4) |
| Joined deworming programs in the last 12 months | Yes | 384 (85.1) |
| | No | 67 (14.9) |
| Other infectious diseases | *Household members with diarrhea in the last 12 months* | |
| | Adults | 423 (93.8) [b] |
| | Children | 1081 (51.4) [b] |
| | *Household members who treated diarrhea in the last 12 months* | |
| | Adults | 396 (93.6) [a] |
| | Children | 1052 (97.3) [a] |
| | *Household members with meningitis in the last 12 months* | |
| | Adults | 6 (1.3) [b] |
| | Children | 58 (92.7) [b] |
| | *Household members who treated meningitis in the last 12 months* | |
| | Adults | 2 (33.3) [a] |
| | Children | 35 (60.3) [a] |
| | *Household members with tuberculosis in the last 12 months* | |
| | Adults | 13 (92.9) [b] |
| | Children | 36 (1.7) [b] |
| | *Household members who treated tuberculosis in the last 12 months* | |
| | Adults | 12 (92.3) [a] |
| | Children | 33 (91.7) [a] |
| | *Household members with pneumonia in the last 12 months* | |
| | Adults | 5 (1.1) [b] |
| | Children | 10 (0.5) [b] |
| | *Household members who treated pneumonia in the last 12 months* | |
| | Adults | 3 (60.0) [a] |
| | Children | 8 (80.0)[a] |
| | *Household members with rabbies in the last 12 months* | |
| | Adults | 0 (0.0) [b] |
| | Children | 12 (0.6) [b] |
| | *Household members who treated rabbies in the last 12 months* | |
| | Adults | 0 (0.0) [a] |
| | Children | 12 (100) [a] |
| | *Household members with measles in the last 12 months* | |
| | Adults | 10 (2.2) [b] |
| | Children | 116 (5.5) [b] |
| | *Household members who treated measles in the last 12 months* | |

| | | |
|---|---|---|
| | Adults | 10 (100) [a] |
| | Children | 107 (92.2) [a] |
| | *Household members with lymphatic filariasis in the last 12 months* | |
| | Adults | 8 (1.8) [b] |
| | Children | 4 (0.2) [b] |
| | *Household members who treated lymphatic filariasis in the last 12 months* | |
| | Adults | 8 (100 )[a] |
| | Children | 4 (100) [a] |
| Vaccination received in last 3 years | *VAT1* | |
| | Adults | 285 (63.2) [b] |
| | Children | 850 (40.4) [b] |
| | *VAT2* | |
| | Adults | 256 (56.8) [b] |
| | Children | 65 4(31.1) [b] |
| | *VAT3* | |
| | Adults | 114 (25.3) [b] |
| | Children | 482 (22.9) [b] |
| | *VAT4* | |
| | Adults | 90 (19.9) [b] |
| | Children | 305 (14.5) [b] |
| | *VAR* | |
| | Adults | 91 (20.2) [b] |
| | Children | 382 (18.1) [b] |
| | *VAA* | |
| | Adults | 98 (21.7) [b] |
| | Children | 365 (17.3) [b] |
| | *BCG* | |
| | Adults | 300 (66.5) [b] |
| | Children | 881 (41.9) [b] |
| Malaria | Number of times experienced in last 12 months | 2373 (-) |
| | Number of times treated in the last 12 months | 2043 (86.1)[a] |
| Water treatment | Yes | 394 (87.4) |
| | No | 57 (12.6) |

[a]: percentage of experience; [b]: percentage of population group
